# Combination of Chenpi and Banxia alleviates synovial inflammation in a rat model of rheumatoid arthritis by targeting the JNK/AP-1 signaling pathway

**DOI:** 10.3389/fimmu.2026.1889598

**Published:** 2026-09-11

**Authors:** Wenxin Zhang, Yue Li, Xiaoyu Zhang, Jie Zhang, Yue Du, Xu Han, Ronghao Cui, Yufeng Li, Fanyan Meng, Di Meng

**Affiliations:** 1 Liaoning University of Traditional Chinese Medicine, Shenyang, Liaoning, China; 2 First Clinical College, China Medical University, Shenyang, Liaoning, China; 3 Department of Chinese Medicine, National Regional Diagnosis and Treatment Center for Traditional Chinese Medicine (TCM) Rheumatology, The First Hospital of China Medical University, Shenyang, Liaoning, China; 4 Shengjing Hospital of China Medical University, Shenyang, Liaoning, China

**Keywords:** Chenpi-Banxia, inflammation, JNK/AP-1, leptin, rheumatoid arthritis

## Abstract

**Introduction:**

Chenpi (*Citrus reticulata*) has both medicinal and dietary applications, while Banxia (*Pinellia ternata*) is frequently combined with Chenpi to treat inflammatory diseases. This study aimed to investigate the potential therapeutic targets and pharmacological mechanisms of the Chenpi-Banxia (CB) herbal combination in rheumatoid arthritis (RA).

**Methods:**

A collagen-induced arthritis (CIA) rat model was used to evaluate the effects of CB on arthritis symptoms, histopathology, and serum cytokine levels. Network pharmacology was applied to identify active compounds and potential targets. Key components were analyzed by high-performance liquid chromatography, and molecular docking and molecular dynamics simulations were performed to assess their interactions with JNK proteins. The regulatory effects of CB on the JNK/AP-1 pathway were further examined in leptin-induced fibroblast-like synoviocytes (FLSs) and CIA rats.

**Results:**

CB significantly reduced paw swelling and arthritis scores, ameliorated joint pathological damage, and decreased TNF-α and IL-6 levels in the serum and synovial tissues of CIA rats. Network pharmacology identified the JNK pathway as a key target. Among five candidate compounds detected in CB-containing serum, nobiletin showed the largest chromatographic peak area. Molecular docking and dynamics simulations supported stable interactions between nobiletin and JNK1/JNK2. *In vitro*, nobiletin and CB-containing serum inhibited leptin-induced FLS proliferation, migration, inflammatory factor release, JNK phosphorylation, and downstream c-Jun and c-Fos expression. In vivo, CB also suppressed JNK/AP-1 activation in synovial tissue.

**Discussion:**

These findings suggest that CB alleviates experimental arthritis and synovial inflammation, at least partly by suppressing leptin-mediated JNK/AP-1 signaling and abnormal FLS activation, thereby supporting its potential therapeutic relevance in RA.

## 1 Introduction

Rheumatoid arthritis (RA) is a chronic systemic autoimmune disease characterized by synovitis, pannus formation, and progressive destruction of articular cartilage and bone (1, 2). Conventional synthetic disease-modifying antirheumatic drugs (csDMARDs) remain the first-line treatment for RA; however, some patients experience inadequate responses or treatment-related adverse effects. While the advent of targeted biologic therapies has revolutionized RA management, studies consistently report suboptimal responses to biologic and targeted synthetic DMARDs (3–5), leaving persistent synovial inflammation as an unmet therapeutic challenge. In this context, natural products and dietary bioactive compounds have received increasing attention as potential sources of complementary anti-inflammatory agents (6).

Obesity has been associated with increased disease activity and reduced responses to biologic and targeted synthetic disease-modifying antirheumatic drugs in some patients with RA, partly through metabolic and immune dysregulation (7, 8). As a key adipokine, leptin, which is secreted by adipocytes, contributes to the pathogenesis of RA and is often found to be elevated in the serum and synovial fluid of RA patients (9, 10). Upon binding to the leptin receptor (LepR), leptin activates several intracellular signaling pathways, including JAK/STAT, PI3K/Akt, and mitogen-activated protein kinase pathways such as ERK and JNK (11, 12). Among these pathways, JNK activation promotes the production of inflammatory mediators, including IL-6 and TNF-α, thereby contributing to inflammatory responses in RA (13, 14). These findings suggest that leptin may serve as an important molecular link between obesity-associated metabolic inflammation and persistent synovial inflammation in RA. Fibroblast-like synoviocytes (FLSs) are major effector cells in RA synovial pathology. Activated RA-FLSs exhibit excessive proliferation, migration, invasion, and secretion of inflammatory mediators and matrix-degrading enzymes. Following activation, JNK phosphorylates c-Jun and enhances the transcriptional activity of activator protein-1 (AP-1), a transcription factor complex mainly composed of Jun and Fos family proteins. The JNK/AP-1 pathway regulates the expression of inflammatory cytokines and matrix metalloproteinases, thereby contributing to persistent synovial inflammation, extracellular matrix degradation, and joint destruction. Therefore, the leptin-mediated JNK/AP-1 axis may link obesity-associated metabolic inflammation with abnormal FLS activation in RA. However, whether CB alleviates synovial inflammation and FLS dysfunction by regulating this signaling axis remains unclear, providing the rationale for selecting the JNK/AP-1 pathway as the mechanistic focus of the present study.

Traditional Chinese Medicine (TCM) has a long-standing history of clinical application and is regarded as a valuable resource for the development of natural therapeutics. In traditional Chinese medicine theory, “phlegm-dampness” accumulation is closely related to obesity and metabolic disorders. Chenpi, the dried pericarp of Citrus reticulata Blanco, is widely used in TCM and as a food ingredient in China (15). Chenpi contains several classes of bioactive constituents, including volatile oils, flavonoids, alkaloids, and polysaccharides, which have demonstrated anti-inflammatory, antioxidant, and metabolic regulatory activities (15, 16). Banxia, the dried tuber of *Pinellia ternata* (Thunb.), Breit, is a plant belonging to the Araceae family. The synergistic combination of the CB herb pair demonstrates dual anti-inflammatory and phlegm-resolving effects in TCM. This combination has been used for thousands of years in China with proven efficacy (17). Furthermore, when used together, Banxia more effectively improves the absorption of active components from Chenpi in damaged tissues, compared to the use of Chenpi alone (18). As a classic “drying phlegm and resolving dampness” medicinal formula, CB’s traditional efficacy aligns with the theoretical framework of traditional Chinese medicine, which is consistent with the regulation of metabolic inflammation caused by “phlegm and dampness”. Our preclinical investigations in collagen-induced arthritis (CIA) models revealed CB’s ability to ameliorate synovial pathology (19). Nevertheless, the representative bioactive constituents, therapeutic targets, and molecular pathways responsible for the effects of CB on RA remain incompletely understood.

This study explored the mechanisms of CB through systematic experimental methods aimed at identifying the therapeutic targets and key active components for treating RA. Accordingly, the present study integrated a CIA rat model, network pharmacology, HPLC analysis, molecular docking, molecular dynamics simulations, and experimental validation in primary rat FLSs. We aimed to identify representative bioactive constituents and potential therapeutic targets of CB and to determine whether CB attenuates synovial inflammation and abnormal FLS activation through regulation of the leptin-mediated JNK/AP-1 signaling pathway.

## 2 Materials and methods

### 2.1 Rat CIA model creation, drug administration, and efficacy assessment

CB (batch numbers: 2301048301, 21083483) was purchased from The First Hospital of China Medical University (Shenyang, Liaoning, China). Thirty-six Sprague-Dawley (SD) rats (weight, 200 ± 10 g, age, six weeks) were obtained from Beijing HuaFuKang Biological Science and Technology Co., Ltd. (Beijing, China), with license number SCXK (Jing) 2019-0008. After one week of adaptive feeding, the rats were randomly divided into six groups: Control (CON), CIA model (CIA), Low-dose CB (CB-L, 1.3 g/kg·d), Medium-dose CB (CB-M, 2.6 g/kg·d), High-dose CB (CB-H, 6.5 g/kg·d), and Methotrexate (MTX, 2 mg/kg·w).

**Figure 1:**
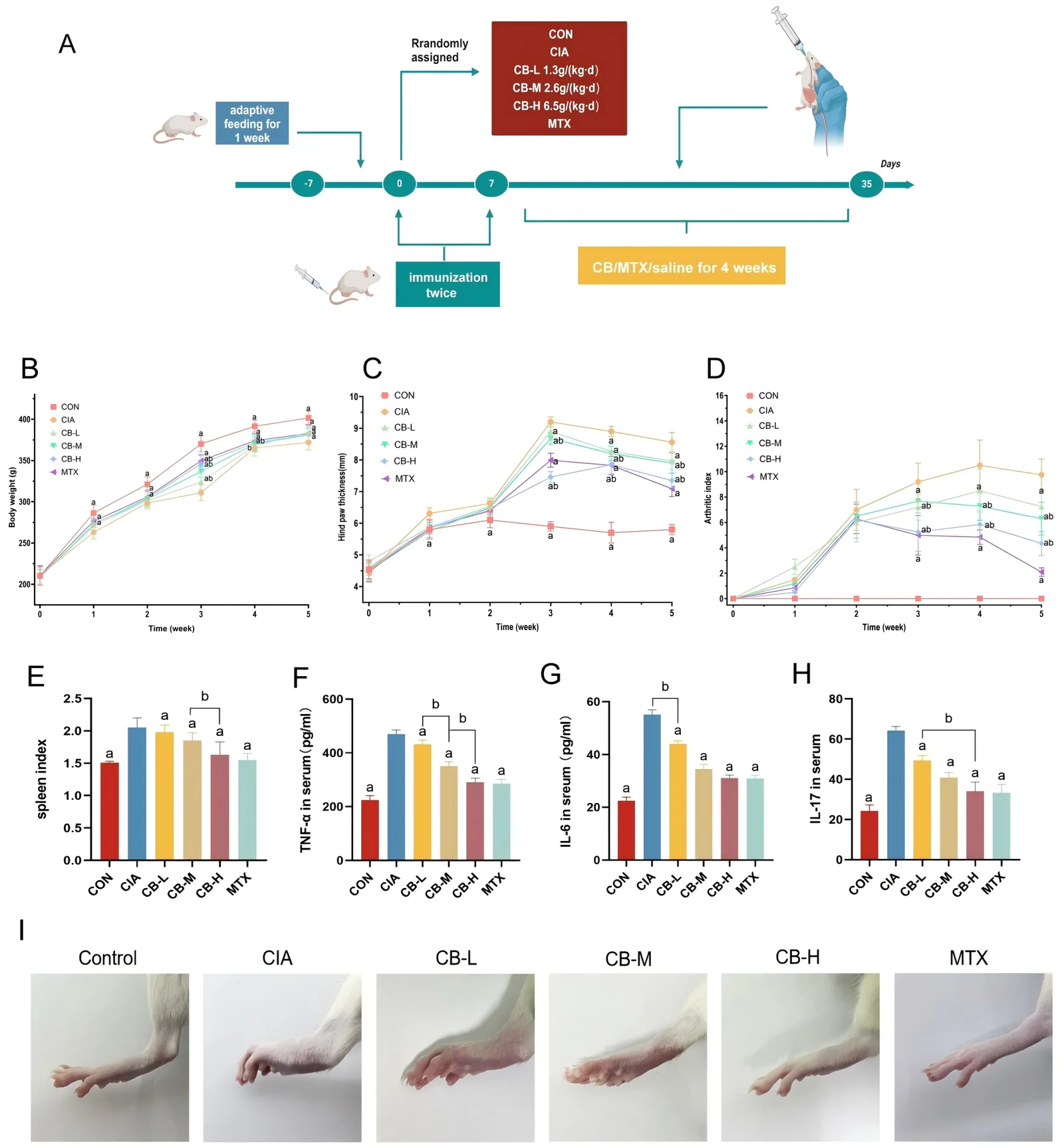
Chenpi-Banxia alleviates arthritis in CIA rats. **(A)** Experimental timeline for CIA. **(B)** Body weight. **(C)** Hind paw thickness. **(D)** Arthritis index. **(E)** Spleen index. **(F–H)** Serum expression levels of TNF-α **(F)**、IL-6 **(G)** and IL-17 **(H)** in CIA rats. **(I)** Representative images of paw appearance 28 days after the first immunization in rats. Data are expressed as mean ± SD (n=3). a: compared with the CIA group, p < 0.01; b: comparison between the two selected groups, p < 0.01.

Except for the CON group, all other groups received an initial immunization for CIA induction, followed by a booster immunization 7 days later. The clinical daily dose of CB was based on a routine prescription containing 16 g of Chenpi and 8 g of Banxia for a 60-kg adult. The total daily dose was therefore 24 g, corresponding to 0.4 g/kg/day, with Chenpi and Banxia combined at a fixed mass ratio of 2:1.Using the Meeh-Rubner formula and the method for converting body weight to body surface area, the dosage of the suspension solution to be administered intragastrically to the rats was calculated. A human to rat dose conversion factor of 6.3 was applied. Accordingly, the rat equivalent dose was calculated as 0.4 g/kg/day × 6.3 = 2.52 g/kg/day and was rounded to 2.6 g/kg/day. Subsequently, low, medium, and high doses of CB were administered intragastrically twice daily at 0.5, 1, and 2.5 times the equivalent dose (2.6 g/kg), respectively. The MTX group received MTX at a dose of 2 mg/(kg·w) via gastric lavage every three days. The CON and CIA groups received an equal volume of physiological saline by oral gavage. The administration volume was 2 mL per rat, and all treatments continued for 28 days (**Figure 1A**).

The animals body weight, right and left hind paw thicknesses, and arthritis index scores were measured weekly, starting from day 0. Briefly, the rats were labeled at the site of their metatarsals, and the thickness of their right and left hind foot metatarsals was measured by marking the same location on the metatarsal on day 0. Meanwhile, the rat arthritis index was evaluated using a scale with scores ranging from 0 to 4, which corresponded to the following: 0, no erythema or swelling in the paw; 1, mild erythema and swelling in the localized toe joints; 2, erythema and swelling in the localized toe joints and toes; 3, erythema and swelling in the paw below the ankle joints; and 4, erythema, pus, purplish discoloration, and severe deformity of the entire paw and ankle joints.

### 2.2 Histological evaluation

Following muscle tissue excision, the right ankle joints of the rats were collected and fixed in a 4% paraformaldehyde solution for seven days. The samples were then decalcified in a 10% EDTA solution (Yuanye, Shanghai) for 28 days, dehydrated, embedded in paraffin, sliced into 5-μm sections, and stained with Hematoxylin and Eosin (HE) (20). The histological evaluation focused on synovial inflammation, cartilage damage, and bone destruction. Two independent observers scored each parameter using a scale with scores ranging from 0 to 3, as described in previous research (21, 22).

### 2.3 Preparation of CB-containing serum

The rats were placed in an induction chamber and anesthetized with isoflurane inhalation (3%, with O_2_ as the carrier gas). Then, the anesthesia was maintained with a face mask (1% isoflurane). Once the deep anesthesia criteria (disappearance of righting reflex and toe clamping pain reflex) were met, the abdomen was incised along the midline, and blood was collected from the abdominal aorta. The blood was kept at room temperature for 2 hours, then centrifuged at 3500 rpm for 15 minutes at 4 °C. The supernatant was carefully collected and stored at -80 °C for further analysis.

### 2.4 HPLC analysis of CB and CB-containing serum

#### 2.4.1 Preparation of CB samples

Chenpi and Banxia granules were obtained from the Department of Pharmacy, the First Hospital of China Medical University. The granules were mixed at a weight ratio of 2:1 and dissolved in sterile distilled water with thorough mixing until completely dissolved. The resulting CB solution was prepared at a final crude-drug concentration of 0.49g/mL. An aliquot of the prepared CB solution (1 mL) was mixed with 3 mL of absolute ethanol, vortexed for 3 min, and sonicated in an ice-water bath for 10 min. After standing at room temperature for 12 h, the mixture was centrifuged at 7,000 × g for 20 min at 4 °C. The supernatant was collected and evaporated to dryness under a gentle stream of nitrogen. The residue was reconstituted with ultrapure water to a final volume of 1 mL and passed through a 0.22μm membrane filter before HPLC analysis.

#### 2.4.2 Preparation of serum samples

CB-containing serum was collected and prepared as described in Section 2.3. An aliquot of CB-containing serum (75 μL) was mixed with 300 μL of precooled methanol at a serum-to-methanol volume ratio of 1:4. The mixture was vortexed for 1 min, sonicated in an ice-water bath for 20 min, and incubated at −20 °C for 1 h to precipitate serum proteins. The samples were then centrifuged at 16,000 × g for 20 min at 4 °C. The supernatant was collected and evaporated to dryness under a gentle stream of nitrogen. The dried residue was reconstituted in methanol and prepared for HPLC analysis.

#### 2.4.3 Preparation of reference standard solutions

Appropriate amounts of the reference standards were separately dissolved in methanol and sonicated for 30 min to ensure complete dissolution. The resulting solutions were passed through a 0.22 μm membrane filter before HPLC analysis.

#### 2.4.4 HPLC conditions

CB samples and CB-containing serum were analyzed using an HPLC system equipped with a diode-array detector and an Agilent Zorbax C18 column (4.6 mm × 250 mm, 5 μm). Acetonitrile and 0.1% aqueous phosphoric acid were used as the mobile phases at a flow rate of 1.0 mL/min. The column temperature was maintained at 30 °C, and chromatograms were recorded at 210, 230, 254, and 280 nm. The injection volume was 10 μL.

### 2.5 Network pharmacology analysis

#### 2.5.1 Collection and screening of CB constituents

Chemical composition Screening of CB: We searched the Traditional Chinese Medicine Systems Pharmacology Database and Analysis Platform (https://www.ncbi.nlm.nih.gov/pccompound/) to obtain the chemical constituents of CB. The constituents were screened based on the Absorption, Distribution, Metabolism, and Excretion (ADME) parameters. The screening conditions included drug Oral Bioavailability (OB) ≥ 30% and Drug-Likeness (DL) ≥ 0.20. The PubChem CIDS and their molecular weights were obtained from the PubChem Compound Database (National Library of Medicine) and used to determine the constituents’ canonical SMILES structures.

#### 2.5.2 Prediction of compound-related targets

The canonical SMILES structures of the chemical constituents obtained from PubChem were submitted to the SwissTargetPrediction database (http://www.swisstargetprediction.ch/), with the species set to Homo sapiens, to predict their potential targets. To identify targets associated with rheumatoid arthritis (RA) development, five comprehensive databases were retrieved using “Rheumatoid Arthritis” as the primary keyword: GeneCards (https://www.genecards.org/), Online Mendelian Inheritance in Man (OMIM, https://omim.org/), the Pharmacogenomics Knowledge Base (PharmGKB, https://www.pharmgkb.org/), the Therapeutic Target Database (TTD, http://db.idrblab.net/ttd/), and DrugBank (https://www.drugbank.ca/).

The retrieved disease targets were merged, and duplicate entries were eliminated. Subsequently, all target names were standardized and corrected using the UniProt Knowledgebase (https://www.uniprot.org/). Finally, the overlapping genes between the compound targets and RA-related targets were identified as potential therapeutic targets of CB using the OmicShare platform (https://www.omicshare.com/tools/), and the corresponding key active components were determined.

#### 2.5.3 GO and KEGG enrichment analyses

Enrichment Analysis of Common Targets: The common targets were analyzed for Gene Ontology (GO) and Kyoto Encyclopedia of Genes and Genomes (KEGG) enrichment using the DAVID 6.8 database (https://davidbioinformatics.nih.gov/), with p < 0.05 as the threshold for significant gene enrichment. Bar graphs were generated and sorted according to the adjusted P-value (P.adjust).

#### 2.5.4 PPI network analysis and network construction

A protein–protein interaction (PPI) network was constructed to characterize the interactions among the common targets (23). The common targets of CB against RA were submitted to the STRING database (version 11.0) to construct the PPI network (24), which was subsequently visualized using Cytoscape 3.6.1 (25). Degree centrality (DC), betweenness centrality (BC), and closeness centrality (CC) were calculated for all nodes in the PPI network using the Cytoscape plug in CentiScaPe, and the mean values of DC, BC, and CC were subsequently determined. A target was retained as a key target only when it simultaneously met all three criteria: (i) its DC value was greater than twice the mean DC value; (ii) its BC value was greater than the mean BC value; and (iii) its CC value was greater than the mean CC value. Targets that failed to meet any one of these criteria were excluded from the key target set.

Cytoscape was also used to construct the key chemical constituent–target network. In the network, nodes of different colors represented the key chemical constituents and targets, whereas edges represented the associations between them. A higher degree value indicated that a node had more connections with other nodes and occupied a more important position in the network.

### 2.6 Molecular docking and core component analysis

The key active ingredients of CB and the core target were selected for molecular docking experiments. First, the crystal structure of JNK was obtained from the Protein Data Bank (http://www.rcsb.org/pdb). The JNK1 crystal structure was selected based on the following criteria: (i) high resolution (≤ 2.0 Å) to ensure accurate atomic coordinates; (ii) human origin (Homo sapiens) to maintain physiological relevance; (iii) structural completeness, i.e., coverage of the entire ATP-binding pocket and key catalytic residues; (iv) absence of mutations, deletions, or unnatural modifications; (v) co-crystallization with an ATP-competitive inhibitor to favor an active, ligand-amenable conformation. Applying these filters to the Protein Data Bank yielded PDB ID 4YR8 as the optimal representative of the human JNK1 catalytic domain. AutoDock Vina 1.5.6 was used to determine the conformational relationships between the protein and drug. The docking results were then visualized and analyzed using PyMOL Viewer 1.5 software, adjusted at appropriate angles, and the interactions between ligands and receptors were analyzed.

### 2.7 Molecular dynamics simulations

Following molecular docking, 100 ns molecular dynamics (MD) simulations of the complexes were performed using GROMACS 2022 software used for the protein, while GAFF2 was applied for the ligand (26). The TIP3P water model was employed to solvate the protein-ligand system and to create a water box with a periodic boundary of 1.2 nm (27). Electrostatic interactions were handled using the Particle Mesh Ewald (PME) method, and the Verlet algorithm was used for nonbonded interactions. The system underwent 100,000 steps of equilibration in both isothermal-isovolumetric and isothermal-isobaric ensembles, with a coupling constant of 0.1 ps and a duration of 100 ps. Van der Waals and Coulomb interactions were calculated with a 1.0 nm cutoff. Finally, the system was simulated at a constant temperature of 310 K and constant pressure of 1 bar for a total of 100 ns.

### 2.8 Cell preparation, drug administration, and JNK pathway expression assessment

Extraction of Rat FLSs: First, the bilateral knees of isolated male SD rats (aged six weeks) were sterilized by immersion in 0.2% benzalkonium bromide for 15 minutes. The synovial tissue from the knee joints were then carefully dissected, sectioned into 1-1.5 mm slices and spread evenly on the bottom of the cell culture dish. The culture dish was placed vertically in a CO2 incubator (at 37 °C and 5% CO2) for 3–4 hours to allow the tissue to adhere to the surface. The tissue blocks were then placed horizontally in dishes and incubated in 4 mL of complete medium containing 20% fetal bovine serum (FBS), 100 U/mL penicillin, and 100 μg/mL streptomycin. On days 3-5, the cells were monitored for migration or outgrowth. Once the FLSs began to migrate from the tissue block, it was removed, and the culture was continued. The medium was changed the following day, and the primary cells were passaged through 3–8 generations and stored for subsequent experiments.

The experiments included seven groups: Control (CON), Model (LEP, 100 ng/mL leptin for 24 h), Low-dose CB-containing serum (CB-L, LEP + 10% CB-L), Medium-dose CB-containing serum (CB-M, LEP + 10% CB-M), High-dose CB-containing serum (CB-H, LEP + 10% CB-H), JNK inhibitor (SP600125, LEP + 15 μM SP600125; **Supplementary Figure 1**) and Nobiletin (NOB, LEP + 25 μM nobiletin; **Supplementary Figure 2**). The serum volume concentration for each group was strictly controlled at 10%, using blank serum to ensure homogeneity.

The SD rats used in the experiments were divided into four groups: Blank (received an equal volume of saline), low-dose CB (CB-L, 1.3 g/kg), medium-dose CB (CB-M, 2.6 g/kg), and high-dose CB (CB-H, 6.5 g/kg) (n=6). Each rat was administered with 2 mL of the drug per administration. These treatments were continued for seven days, and blood samples were collected from the abdominal aorta 1 hour after the final administration. The blood was then centrifuged at 3000 rpm for 10 minutes to separate the serum, which was collected and filtered through a 0.22 μm membrane. The serum was inactivated by incubating at 56 °C for 30 minutes.

The Cell Counting Kit-8 (CCK-8) assay was used to determine the optimal concentration and timing of leptin and CB intervention in FLSs. First, FLSs were seeded at a density of 1 × 10^5 cells/mL in 96-well plates and incubated overnight. After 24 hours of treatment with leptin at concentrations of 25, 50, and 100 ng/mL for the low, medium, and high concentration groups, respectively, the incubation was terminated. The blank and negative control groups were incubated with 10% FBS. Subsequently, 10 μL of CCK-8 solution was added to each well, followed by a two-hour incubation period. Absorbance (A) was measured at 450 nm using a microplate reader.

After determining the optimal concentration and timing of leptin treatment, different concentrations of the CB-containing serum (2.5%, 5%, 10%, 15%, and 20%) were added. After a 24-hour treatment period, the CCK-8 assay was used to evaluate the effects of different CB-containing serum concentrations on FLSs viability and to determine the optimal concentration of CB-containing serum administration. Cell viability was calculated using the following formula: (A_experimental group - A_blank group)/(A_control group - A_blank group)× 100%.

For the wound healing assay, FLSs were seeded in 6-well plates at a density of 5 × 10*5 cells/well and incubated overnight to establish a confluent monolayer (100% confluence). A pipette tip was then used to create a uniform wound across the monolayer, and the initial wound gap was photographed immediately (at time 0 h). The cells were subsequently washed treated with the desired experimental conditions. After a 24-hour incubation, the wound gap was photographed again to assess healing progress. The entire experimental procedure was repeated three times to ensure reproducibility and statistical significance.

For the cell proliferation assay, FLSs were seeded at a density of 1 × 10^4 cells/mL and cultured until adherence in a high-glucose medium containing 5% FBS. Three replicate wells were set up for each group. After the designated treatment, 10 μL of CCK-8 solution and 90 μL of basal medium were added to each well of the culture plate. The plate was then incubated for an additional 1.5 hours. Absorbance (A) was measured at 450 nm using a microplate reader to quantify cell viability and proliferation.

### 2.9 Immunofluorescence assay

*In vivo* and *in vitro* immunofluorescence (IF) analyses were performed to identify cells and detect the expression of JNK pathway-related proteins. First, cells were evenly spread in confocal petri dishes for observation. The cells were then washed and fixed. Next, paraffin sections were prepared, followed by dewaxing and hydration. The sections and cells were permeabilized with 0.1% Triton X-100 on ice and blocked with 5% Bovine Serum Albumin (BSA) for 60 minutes. Subsequently, the sections and cells were incubated with primary antibodies (Vimentin ab92547, JNK ab179461, p-JNK ab124956, and c-Jun ab32137) at a 1:250 dilution overnight at 4°C. Afterward, the sections and cells were incubated with fluorescent secondary antibodies (Goat Anti-Rabbit IgG H&L [DyLight^®^ 488, ab96883] at a 1:1000 dilution for 1 hour (shown in green). Nuclear DNA was stained with DAPI, resulting in blue labeling. Finally, images were captured using a confocal microscope (Leica Microsystems, TCSSP8).

### 2.10 Immunohistochemistry

Paraffin-embedded ankle joint sections (4-μm thick) were baked at 60 °C for 90 min, deparaffinized in xylene, and rehydrated through a graded ethanol series. Antigen retrieval was performed using pressure-mediated heating for 3 min in 0.01 M citrate buffer (pH 6.0) for both TNF-α and IL-6. After cooling to room temperature, the sections were washed with PBS and incubated with an endogenous peroxidase blocking reagent for 10 min, followed by blocking with normal nonimmune goat serum for 15 min at room temperature. The sections were then incubated overnight at 4 °C with primary antibodies against TNF-α (1:100 dilution; Cat. No. #AF7014, Affinity Biosciences) and IL-6 (1:100 dilution; Cat. No. #DF6087, Affinity Biosciences). After rewarming at 37 °C for 30 min and washing with PBS, the sections were sequentially incubated with a biotinylated goat anti-mouse/rabbit IgG polymer and streptavidin–horseradish peroxidase for 10 min each at room temperature. Immunoreactivity was visualized using freshly prepared 3,3′-diaminobenzidine (DAB) for 1–3 min under microscopic observation. The sections were counterstained with hematoxylin for 3 min, differentiated in 1% acid alcohol, dehydrated through graded ethanol, cleared in xylene, and mounted with neutral resin. Finally, immunoreactive images were acquired using a light microscope under identical acquisition settings. TNF-α and IL-6 immunoreactivity was quantified using ImageJ software. Multiple independent, non-overlapping fields were analyzed for each sample, and the mean value was used as the representative value for between-group comparisons. The relative protein level was estimated based on the percentage of positively stained area and expressed as Area%.

### 2.11 Enzyme-linked immunosorbent assay

First, blood samples collected from each group of rats were centrifuged to isolate the serum and erythrocytes. After drug treatment, the cell supernatant was collected and centrifuged to remove debris and polymers. Subsequently, the expression levels of TNF-α and IL-6 in the serum and cell supernatant were measured using respective ELISA kits (Rat TNF-α, IL-6 ELISA Assay Kits, Shanghai Guduo Bio-Technology Co., Ltd.), according to the manufacturer’s instructions. All tests were repeated three times.

### 2.12 WB analysis

First, FLSs and tissue blocks were lysed with RIPA lysis buffer containing PMSF (100:1). Subsequently, the supernatant was aspirated after centrifugation to obtain the protein solution. The Bicinchoninic Acid (BCA) assay was then used to determine protein concentration. Equal volumes of total protein were then separated by SDS-PAGE and transferred to Polyvinylidene Difluoride (PVDF) membranes. After blocking with 5% BSA, the membranes were incubated overnight with primary antibodies (JNK, ab179461; p-JNK, ab124956; c-Jun, ab32137; c-Fos, ab302667; and Leptin, ab16227). The membranes were subsequently rinsed several times with TBST before incubation with the secondary antibody, Goat Anti-Rabbit IgG H&L (HRP, ab205718) for 2 hours. The β-tubulin antibody (ab176560, 1/1000) was used as a loading control. The gray values of the protein bands were quantified using ImageJ software.

### 2.13 Statistical analysis

Statistical analyses and graph generation were performed using SPSS 26.0 and Origin Pro 2018 software. One-way analysis of variance (ANOVA) was used for normally distributed data. The Levene’s test for homogeneity of variance was applied to the measured data, with multiple comparisons performed using the Least Significant Difference (LSD) method when variances were homogeneous, and Dunnett’s T3 method when variances were not homogeneous. The non-parametric Kruskal-Wallis test was used for non-normally distributed data. Pearson correlation analysis was applied to compare the two groups. Results with p-value < 0.05 were considered statistically significant.

## 3 Results

### 3.1 Effects of CB on arthritis and inflammation in CIA rats

A CIA rat model was established to investigate the effects of CB on body weight, spleen index, arthritis index, paw swelling, and inflammatory markers. According to the results, rats gradually lost weight and developed swellings in paws, nodules, and erythema 21–35 days after the first injection. Conversely, compared to the CIA group, MTX and CB improved rats’ body weight, paw thickness, and arthritic index scores (**Figures 1B–D**). Furthermore, no significant difference was observed between the MTX and CB groups. Additionally, the spleen index of CIA rats increased significantly after immunization (**Figure 1E**). However, following CB and MTX treatment, the spleen index of CIA rats decreased.

Serum levels of the inflammatory mediators IL-6, TNF-α and IL-17 were assessed by using the ELISA method. CIA rats exhibited significantly higher serum concentrations of IL-6, TNF-α and IL-17 compared to CON group rats, with significant reductions following CB and MTX treatments (*p* < 0.01) (**Figures 1F–H**). **Figure 1I** shows the paw swelling in the rats. Notably, the paw swelling was decreased following treatment compared to the CIA group. Moreover, visual examination revealed that the CB-H and MTX groups decreased the paw swelling to the same degree, comparable to those observed in the CIA rats.

### 3.2 Effects of CB on the histopathology and inflammation of the ankle joint in CIA rats

**Figure 2:**
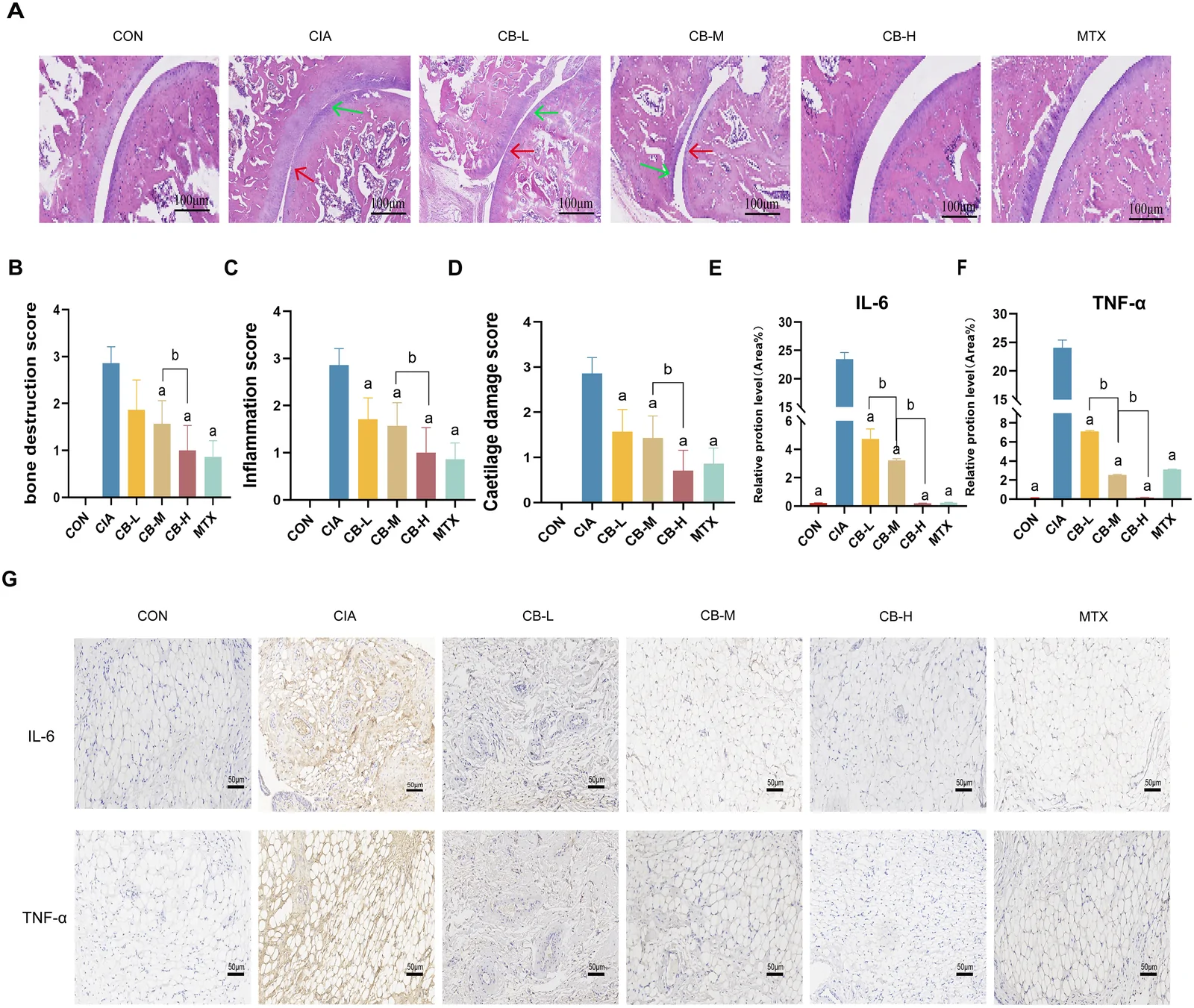
Histopathological changes in CIA rat joints. **(A)** H&E staining of ankle joint tissues (×40). The green arrows indicate inflammatory infiltration, while the red arrows denote bone destruction. **(B)** Bone destruction. **(C)** Synovial inflammation. **(D)** Cartilage damage. **(E, F)** Expression levels of IL-6 **(E)** and TNF-α **(F)** in the synovial tissue of CIA rats. **(G)** IHC analysis of joint synovial tissue. Data are expressed as mean ± SD (n=3). a: compared with the CIA group, *p* < 0.01; b: compared between the two selected groups, *p* < 0.01.

The CB treatment alleviated the histopathological changes in the knee joints of CIA rats. Briefly, tissue sections were prepared 33 days following the initial immunization to examine the histopathological characteristics of the rat ankle joints. HE staining was systematically scored in three aspects: bone destruction, synovial inflammation, cartilage damage (**Figure 2A**). Compared to the CON group, the CIA model group exhibited significantly higher bone destruction, inflammation and cartilage damage scores (*p* < 0.01). Conversely, CB and MTX treatments significantly attenuated rats’ histopathologic scores at different doses (*p* < 0.01) (**Figures 2B–D**). Furthermore, IHC staining showed markedly increased TNF-α and IL-6 immunoreactivity in the synovial tissues of CIA rats. Quantitative analysis demonstrated that the percentages of TNF-α- and IL-6-positive staining areas were significantly higher in the CIA group than in the CON group (*p* < 0.01). Compared with the CIA group, all CB-treated groups and the MTX group showed significantly reduced positive staining areas for both TNF-α and IL-6 (*p* < 0.01). The levels in the CB-L and CB-M groups remained significantly higher than those in the CON group (*p* < 0.01), whereas no significant differences were observed between the CB-H or MTX group and the CON group. Among the CB-treated groups, CB-H showed the lowest mean positive staining area for both inflammatory factors (**Figures 2E, F**).

### 3.3 Prediction of the anti-RA mechanism of action of CB and potential active ingredients based on network pharmacology

A network pharmacology approach was applied to explore the potential mechanisms of action of CB in treatment of RA. Using the TCMSP platform with oral bioavailability(OB) ≥ 30% and drug-likeness(DL) ≥ 0.20 as the screening criteria, 17 active components of CB were identified, as detailed in **Table 1**.

**Table 1:** Major active ingredients of CB against RA and their OB and DL values.

Herb name	MOL ID	Molecule name	OB	DL
Banxia	MOL001755	24-Ethylcholest-4-en-3-one	36.08	0.76
	MOL002670	cavidine	35.64	0.81
	MOL002714	baicalein	33.52	0.21
	MOL002776	baicalin	40.12	0.75
	MOL000358	beta-sitosterol	36.91	0.75
	MOL000449	stigmasterol	43.83	0.76
	MOL005030	gondoic acid	30.7	0.2
	MOL000519	coniferin	31.11	0.32
	MOL006936	10,13-eicosadienoic	39.99	0.2
	MOL006957	(3S,6S)-3-(benzyl)-6-(4-hydroxybenzyl)piperazine-2,5-quinone	46.89	0.27
	MOL003578	Cycloartenol	38.69	0.78
	MOL006967	beta-D-Ribofuranoside, xanthine-9	44.72	0.21
Chenpi	MOL000359	sitosterol	36.91	0.75
	MOL004328	naringenin	59.29	0.21
	MOL005100	5,7-dihydroxy-2-(3-hydroxy-4-methoxyphenyl)chroman-4-one	47.74	0.27
	MOL005815	citromitin	86.9	0.51
	MOL005828	nobiletin	61.67	0.52

**Figure 3:**
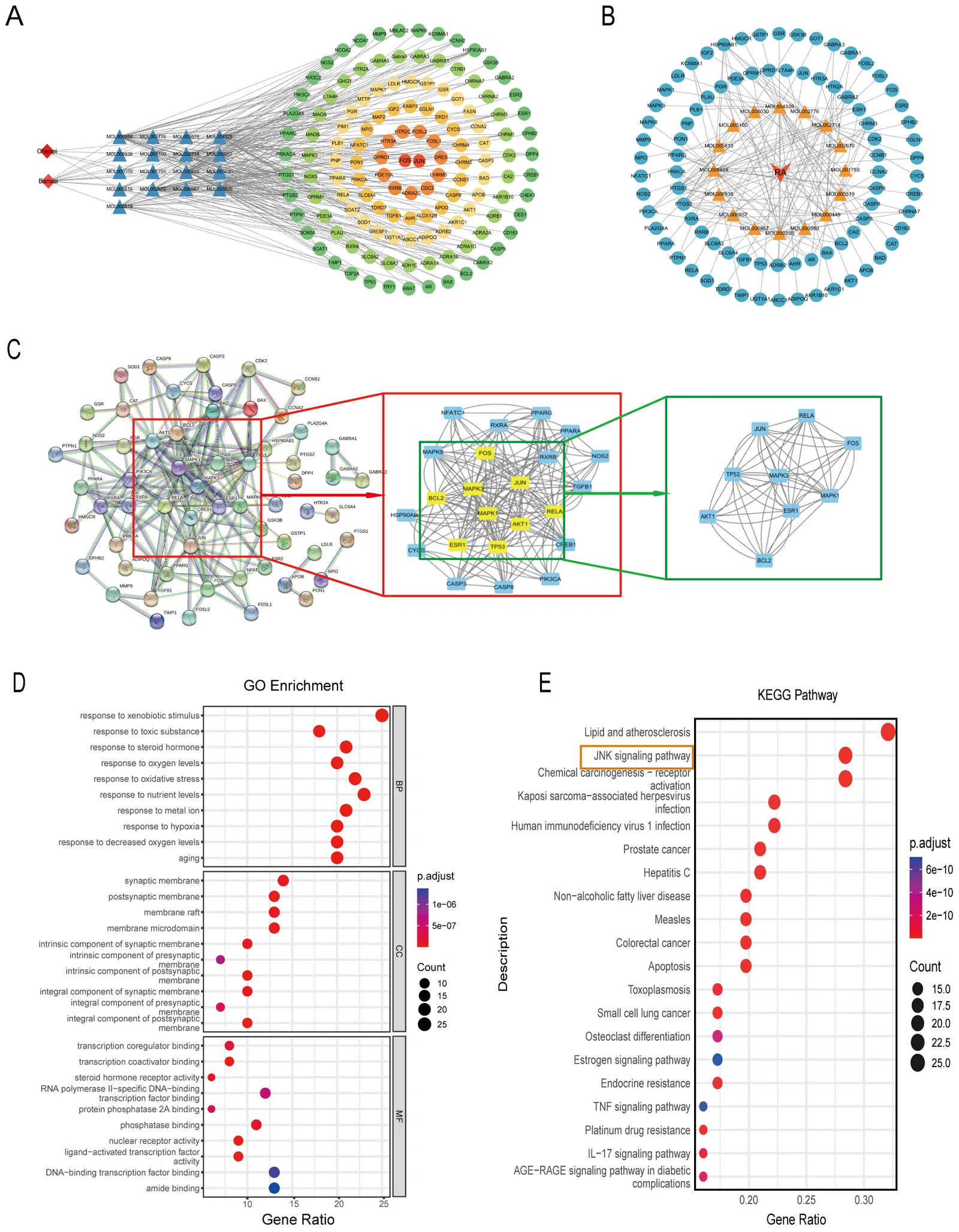
Prediction of the mechanism of action of CB against RA based on network pharmacology. **(A)** The CB herbs-ingredients-target network. **(B)** Ingredients-targets-RA network. **(C)** Interactive PPI network of CB putative targets and RA-related targets; the PPI network of significant proteins obtained from the first network; and the core PPI network of candidate CB targets for RA treatment derived from the second network. **(D)** GO analysis of core candidate targets of CB against RA. The top 10 terms in each GO category with *p* < 0.05 were selected. BP, biological process; CC, cellular components; MF, molecular function. **(E)** KEGG pathway enrichment analysis of core candidate targets of CB against RA. The top 20 pathways with significant enrichment (*p* < 0.05) were identified.

Subsequently, 134 potential targets of CB were predicted. For RA-related target screening, 2,174 targets were retrieved from GeneCards, 326 from OMIM, 142 from PharmGKB, 89 from TTD, and 263 from DrugBank using “Rheumatoid Arthritis” as the keyword. In total, 2,994 target records were retrieved from the five databases before duplicate removal. After merging the datasets, removing duplicate entries, and standardizing the target names, cross-analysis of the compound-related and RA-related target datasets identified 84 overlapping genes as potential therapeutic targets of CB against RA. An herb–ingredient–target network (**Figure 3A**) and an ingredient–target–disease network (**Figure 3B**) were constructed. Based on topological analysis of the PPI network, nine hub genes, including JUN and FOS, were identified (**Figure 3C**). GO and KEGG enrichment analyses were subsequently performed on the common targets. GO analysis indicated that these genes were involved in biological processes related to inflammation, apoptosis, growth, and proliferation. In addition, the common targets were associated with lipid metabolism and nutrient -related processes (**Figure 3D**). KEGG enrichment analysis identified 20 significantly enriched pathways, among which the JNK signaling pathway was notably enriched (**Figure 3E**). Among the significantly enriched pathways, the JNK signaling pathway showed the highest enrichment significance after the broad disease-related pathway “Lipid and atherosclerosis.” In addition, JNK signaling is closely involved in synovial inflammation and inflammatory cytokine production in rheumatoid arthritis and is consistent with the proposed pharmacological effects of the Chenpi–Banxia herbal pair. Therefore, based on both the enrichment significance and its biological relevance to the study hypothesis, the JNK signaling pathway was selected for subsequent mechanistic validation.

### 3.4 Identification of the representative active compound in CB by HPLC

**Figure 4:**
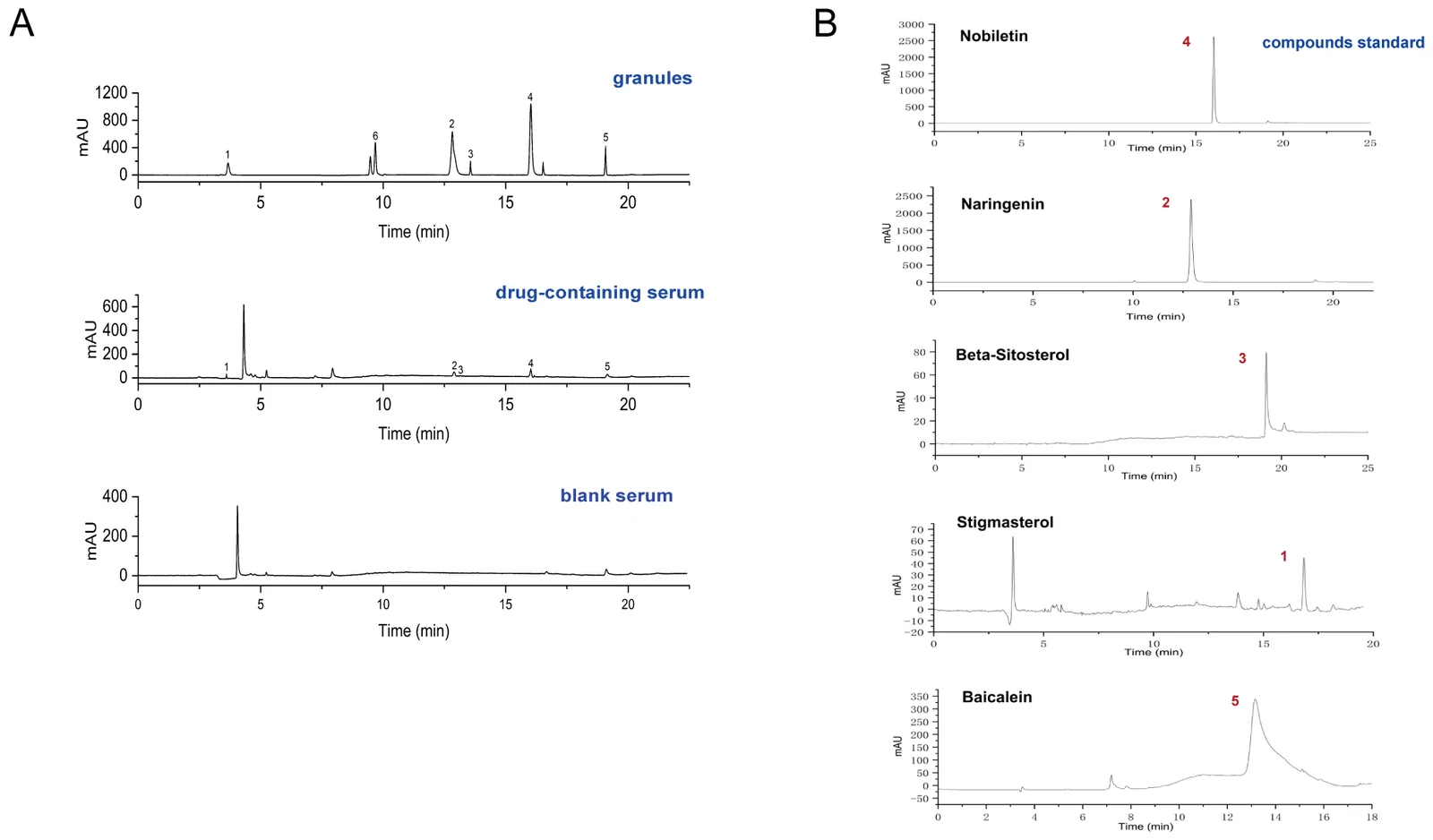
Content of representative active components in CB. HPLC digital fingerprint of CB granules, CB drug-containing serum, blank serum **(A)** and five active compounds **(B)**.

In further analysis, we investigated whether the predicted active ingredients could be absorbed into the bloodstream. The top five active compounds (Stigmasterol, Naringenin, Baicalein, Nobiletin, and Beta-Sitosterol) with the highest DC in the CB-Targets RA network were selected and quantified by HPLC. Blank serum and CB-containing serum samples were prepared and CB granule samples were analyzed by HPLC using 10 μL of each sample. Chromatograms were recorded (**Figures 4A, B**). Similarly, 10 μL of each standard of the main active compounds of CB was injected for analysis. The characteristic peaks in the chromatogram of CB-containing serum, with stable and prominent peaks, were identified and calibrated. Five characteristic peaks corresponding to the retention times of the standard were labeled. Subsequently, similarity calculations were performed, and the results showed similarity values above 0.90. A comparison of the retention times of the peaks with the online mass spectra and authentic standards identified five peaks, including Stigmasterol (peak 1), Naringenin (peak 2), Baicalein (peak 3), Nobiletin (peak 4), and Beta-Sitosterol (peak 5). The peak areas of these five characteristic peaks were then quantified in CB-containing serum. The peak areas and peak heights of the five characteristic peaks in CB-containing serum are presented in **Table 2**. Among the five compounds identified in CB-containing serum, nobiletin was selected for subsequent experimental validation by integrating the network pharmacology and HPLC results. Nobiletin ranked among the candidate compounds with relatively high degree centrality in the compound–target network, suggesting its potential involvement in the regulation of multiple RA-related targets. In addition, its presence in CB-containing serum confirmed that nobiletin could enter the systemic circulation after CB administration. Therefore, based on its network importance and confirmed serum exposure, nobiletin was selected as a representative active constituent of CB for further mechanistic investigation.

**Table 2:** Peak area and peak height of five characteristic peaks in CB-containing serum.

Peak	Name	2D Structure	Peak area mAU*S	Peak height mAU	Retention time min
1	Stigmasterol	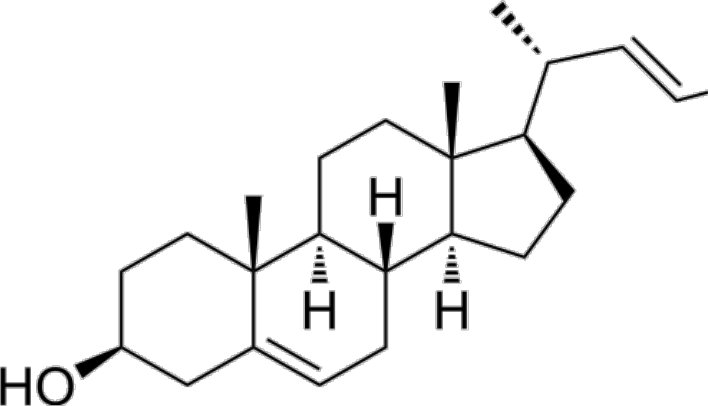	0.188	32.020	3.605
2	Naringenin	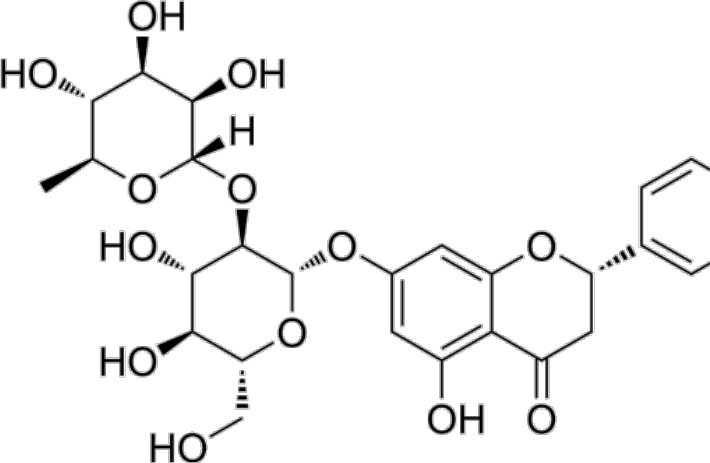	6.975	49.624	12.891
3	Baicalein	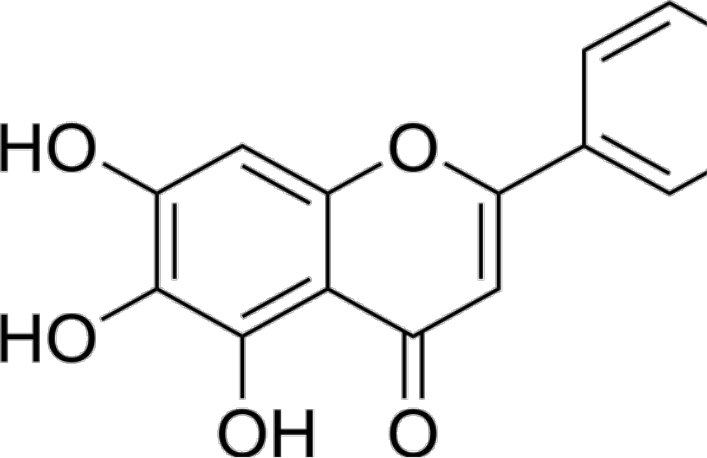	2.567	18.182	13.151
4	Nobiletin	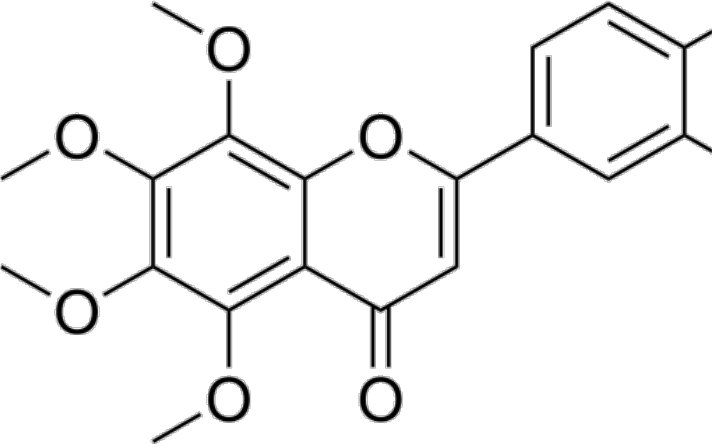	7.286	75.679	16.025
5	Beta-Sitosterol	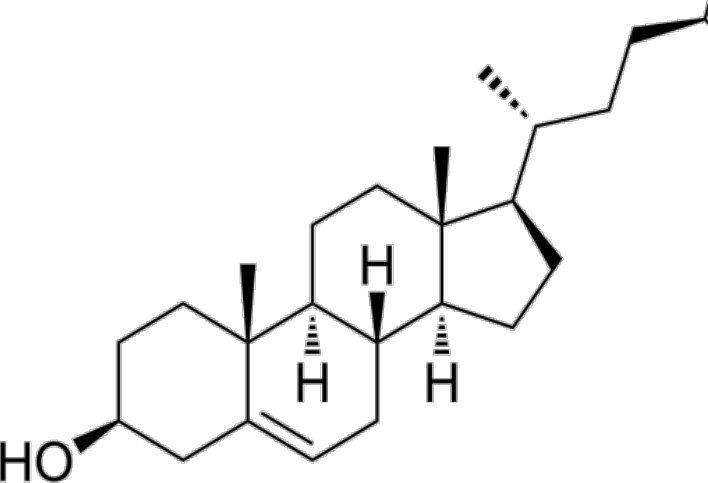	3.886	29.624	19.145

### 3.5 Molecular docking and MDs verified the targets of active compounds in CB

**Figure 5:**
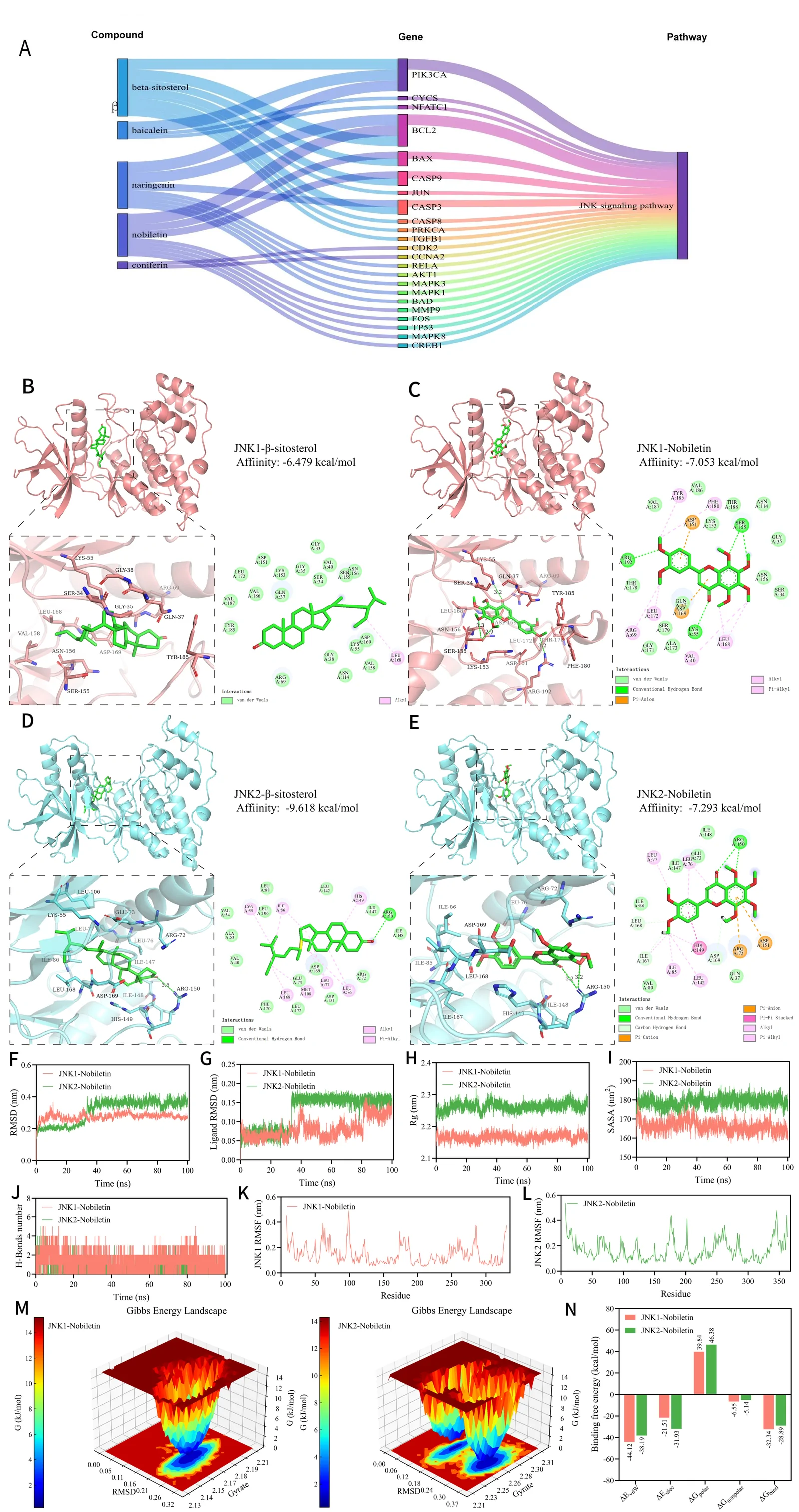
Molecular docking and MDs verified the target of CB. **(A)** Alluvial map of the main active ingredients of CB in RA related to the JNK signaling pathway. The alluvial plot was visualized using the online chiplot tools (https://www.chiplot.online); **(B–E)** 3D interaction diagrams of the core compound with JNK protein, including β-sitosterol - JNK1 **(B)**,nobiletin - JNK1 **(C)**, β-sitosterol - JNK2 **(D)**, nobiletin - JNK2 **(E)**; **(F)** RMSD curve of JNK1 - nobiletin and JNK2 - nobiletin complex with time; **(G)** Ligand RMSD curve of JNK1 - nobiletin and JNK2 - nobiletin complex with time; **(H)** Rg curves of JNK1 - nobiletin and JNK2 - nobiletin complex changes at different times; **(I)** SASA curves of JNK1 - nobiletin and JNK2 - nobiletin complex changes at different times; **(J)** Curves of the number of hydrogen bonds between JNK1 - nobiletin and JNK2 - nobiletin complex at different times; **(K, L)** RMSF curves of amino acid backbone atoms of JNK1 - nobiletin complex at different times. **(L)** RMSF curves of amino acid backbone atoms of JNK2 - nobiletin complex at different times.

Based on HPLC component identification and alluvial plot analysis (**Figure 5A**), molecular docking was performed to predict the binding affinities of two key CB compounds, nobiletin and β-sitosterol, toward JNK1 and JNK2. The predicted binding energies of nobiletin with JNK1 and JNK2 were −7.053 and −7.293 kcal/mol, respectively, whereas those of β-sitosterol with JNK1 and JNK2 were −6.479 and −9.618 kcal/mol, respectively (**Figures 5B–E**). All binding energies were lower than -5 kcal/mol, suggesting favorable predicted interactions between the two compounds and JNK proteins. Nobiletin, with the highest content in CB, emerged as the most promising candidate for further investigation.

To evaluate the stability and dynamics of the nobiletin-JNK complex, molecular dynamics (MD) simulations were conducted over 100 ns. As shown in **Figure 5F**, the RMSD of the JNK1-Nobiletin complex rose rapidly at the beginning of the simulation and then mainly remained at approximately 0.25 - 0.31 nm; the RMSD of the JNK2-Nobiletin complex remained at 0.18 - 0.23 nm for the first approximately 35 ns, then underwent a significant conformational transition and increased to approximately 0.34 - 0.40 nm. These results suggest that nobiletin is highly stable when bound to both JNK1 and JNK2. Further analysis revealed that the rotational radius (Rg) of the JNK1-nobiletin and JNK2-nobiletin complexes remained at approximately 2.15-2.18 nm and 2.24-2.30 nm respectively, indicating that both proteins maintained a relatively stable overall compactness (**Figure 5H**).

The solvent accessible surface area (SASA) of the JNK1-nobiletin and JNK2-nobiletin complexes was mainly distributed at approximately 162–171 nm² and 176–185 nm², suggesting that the complex did not undergo significant expansion or contraction (**Figure 5I**). **Figure 5J** indicated that the number of hydrogen bonds between JNK1-nobiletin complexes typically remained at about 1-3, and sometimes up to 4 or more; while the number of hydrogen bonds in JNK2-nobiletin complexes was smaller and more intermittent. These results suggest that nobiletin may have formed a more stable polar interaction network with JNK1. As shown in **Figures 5K, L**, the root mean square fluctuation (RMSF) values of the JNK1-nobiletin and JNK2-nobiletin complexes were mostly below approximately 0.20 nm, indicating low flexibility and high stability.

In summary, both JNK1-nobiletin and JNK2-nobiletin complexes are stable and show favorable hydrogen bonding interactions, making nobiletin a promising candidate for further research.

The MM/PBSA calculations indicate (**Figure 5N**) that the total binding free energy of both complexes is negative. Specifically, JNK1–nobiletin: -32.34 kcal/mol, JNK2–nobiletin: -28.89 kcal/mol. The JNK1 system exhibits a stronger van der Waals contribution and a lower polar solvation penalty, ultimately resulting in a more negative total binding free energy. This result is consistent with the hydrogen bond analysis, suggesting that nobiletin may form a more persistent binding network within the JNK1 pocket.

### 3.6 Nobiletin and CB inhibit leptin-induced inflammation, proliferation, migration and suppress the JNK/AP-1 pathway in FLSs

**Figure 6:**
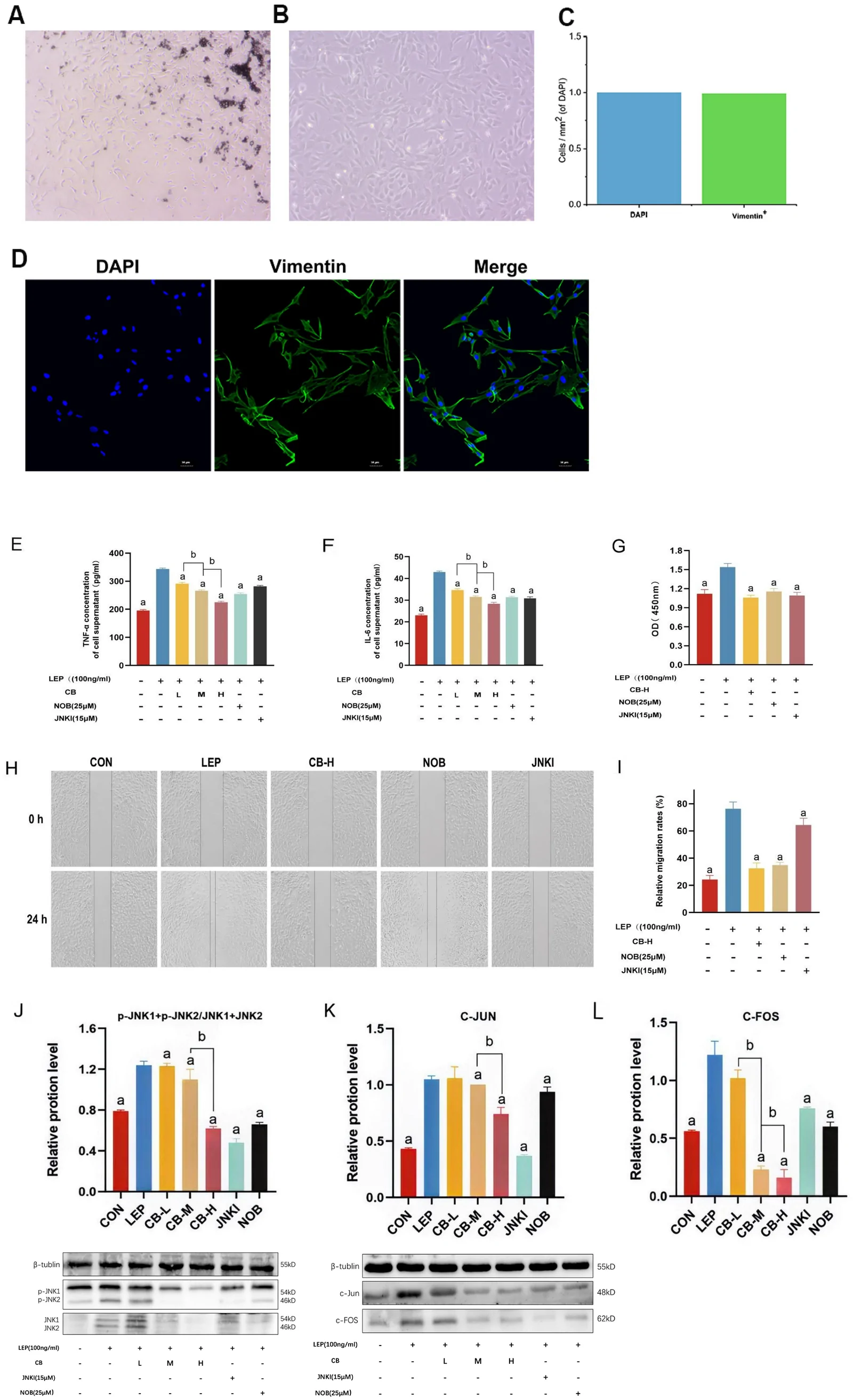
NOB and CB inhibit leptin-stimulated FLSs inflammation, proliferation, migration and suppress the JNK/AP-1 pathway in leptin-induced FLSs (mean ± SD, n = 3). **​(E, F)** Expression levels of TNF-α **(E)** and IL-6 **(F)** in FLS supernatants. **(G)** Absorbance value of cell proliferation assays. **(H)** Wound-healing assay (×40). **(I)** Relative migration rates (%). **(J–L)** Expression levels of p-JNK1+JNK2/JNK1+JNK2 **(J)**, c-JUN **(K)**, and c-FOS **(L)** in FLS. a: compared to the LEP group, *p* < 0.01; b: compared between two selected groups, *p* < 0.01.

To determine the inhibitory effect of CB-containing serum on leptin-induced inflammatory responses in synovial cells, we applied the JNK inhibitors and nobiletin concurrently to elucidate the mechanism of action of the drug. FLSs were co-cultured with leptin at concentrations of 25, 50, and 100 ng/mL for 24 hours (**Supplementary Figure 3**). Compared with the control group, there was no significant change in cell proliferation after 24 hours of treatment with 25 ng/mL leptin, while 50 ng/mL and 100 ng/mL leptin both significantly promoted the proliferation of FLS (*p* < 0.05), and the proliferative effect of the 100 ng/mL group was significantly stronger than that of the 50 ng/mL group (*p* < 0.05). Therefore, 100 ng/mL leptin was selected for the subsequent experiments. Moreover, results of the CCK-8 assays revealed no cytotoxicity for CB-L, -M, and -H serum at concentrations ranging from 0% to 15% over 12 hours (**Supplementary Figure 4**). The data show that there is a non-linear relationship between serum concentration and inhibition rate, and the inhibition rate begins to increase significantly when the concentration exceeds approximately 9.0%. Consequently, 10% CB-containing serum was chosen for subsequent treatments. We further analyzed the morphology of rat primary synovial cells. After the second passage, the FLSs were enlarged and extended (**Figure 6A**). At passages 3 to 8, the FLSs displayed a fibroblast-like morphology (**Figure 6B**). Immunofluorescence analysis demonstrated that over 98% of the cells were positive for vimentin (**Figures 6C, D**), confirming that the cultured cells were FLSs.

As expected, the concentrations of TNF-α and IL-6 were significantly higher in the supernatants of leptin-treated FLSs than in the CON group (*p* < 0.01) (**Figures 6E, F**). CB-containing serum reduced the leptin-induced increases in TNF-α and IL-6 levels in a dose-dependent manner. CB-L, CB-M, and CB-H were initially included to evaluate the *in vitro* dose–response of CB-containing serum. Because CB-H produced the most pronounced inhibitory effects in these initial analyses, it was selected as the representative CB dose for subsequent experiments in which only one CB-containing serum group was included. The NOB group and the JNKI group showed decreased levels of these inflammatory factors (*p* < 0.01). These findings indicated that NOB and CB attenuated the leptin-induced inflammatory response in FLSs. Results of the CCK-8 assay demonstrated that leptin stimulation significantly increased FLSs proliferation (**Figure 6G**). In contrast, NOB, CB, and JNKI treatment more effectively inhibited leptin-induced hyperproliferation compared to the LEP treatment. The results showed that the LEP group had significantly faster cell migration than the CON group after 24 hours of leptin treatment (**Figure 6H**). In contrast, the NOB, CB-H, and JNKI groups showed reduced cell migration compared to the LEP group. Further analysis confirmed that NOB, CB-H and JNKI inhibited leptin-induced FLSs migration (**Figure 6I**).

The effect of NOB and CB-containing serum on the expression of proteins related to the JNK pathway was determined by western blot assay. The data shown in **Figures 6J–L** indicate that the ratio of p-JNK1+JNK2/JNK1+JNK2 phosphorylation and the expression levels of c-JUN and c-FOS were significantly higher in the LEP group compared to the CON group. Both NOB and CB treatments significantly reduced the expression of p-JNK1+JNK2/JNK1+JNK2, c-JUN, and c-FOS compared with LEP. Notably, the effects of CB were dose-dependent. These results demonstrated that NOB and CB inhibited the leptin-mediated JNK phosphorylation and the downstream factor, AP-1. Similar results were observed following treatment with the specific JNK group (*p* < 0.01).

### 3.7 Nobiletin and CB target JNK in leptin-induced FLSs

**Figure 7:**
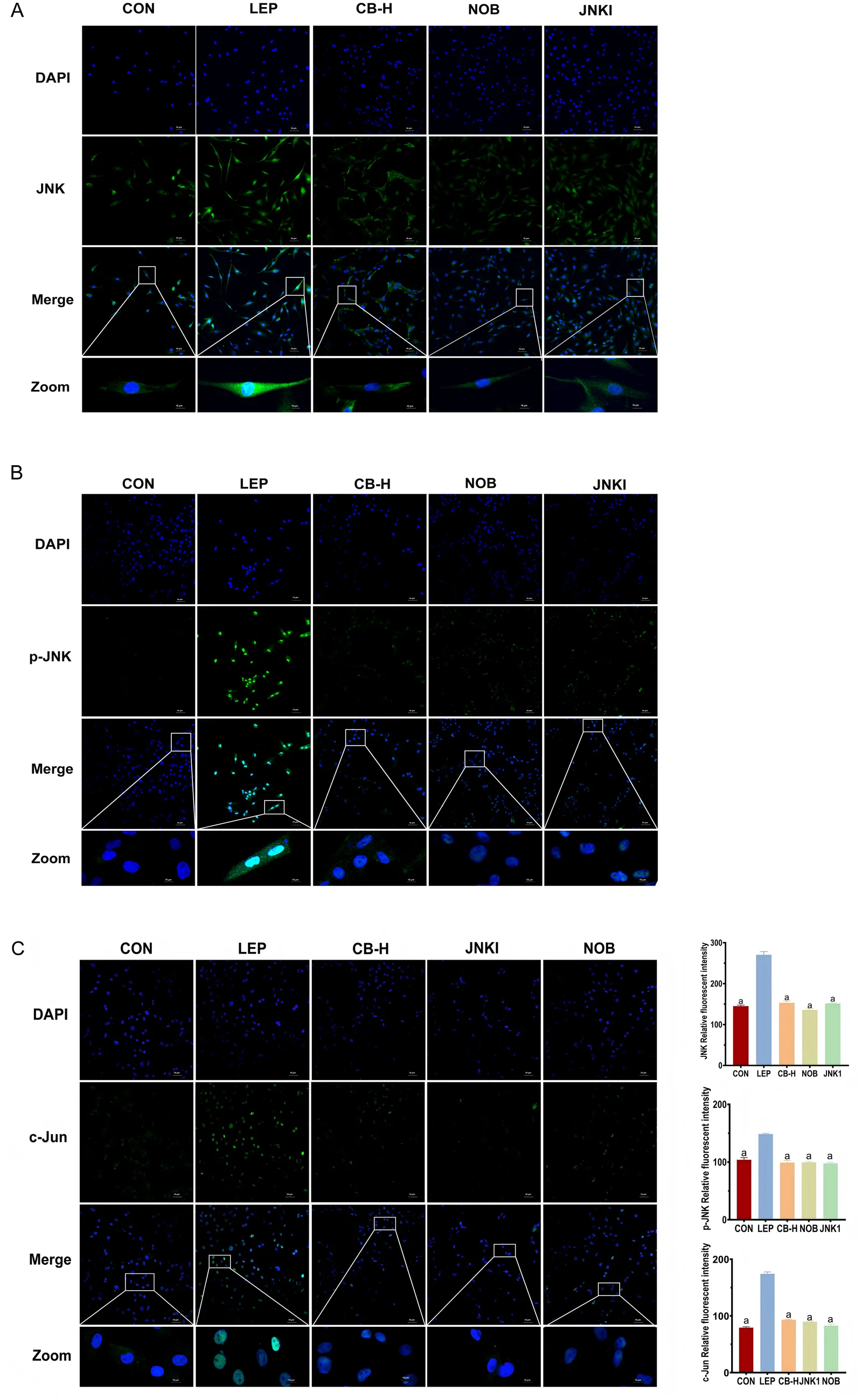
NOB and CB target JNK in leptin-induced FLSs (mean ± SD, n = 3). **(A–C)** Representative fluorescence confocal microscopy images of FLS stained for JNK **(A)**, p-JNK **(B)**, and c-Jun **(C)** (green), and DAPI (blue). Scale bar = 50 μm; zoomed-in scale bar = 10 μm.

Based on the initial dose–response results described in Section 3.6, CB-H produced the most pronounced inhibitory effects and was therefore selected as the representative CB-containing serum group for subsequent mechanistic validation. The effects of NOB and CB-H on JNK signaling molecules in FLSs were subsequently examined using immunofluorescence assays. The effects of NOB and CB on the JNK signaling molecules in FLSs were examined through the immunofluorescence assay. The results indicated that NOB and CB inhibited JNK phosphorylation and nuclear translocation. Analysis of the LEP group revealed a substantial elevation in green fluorescence, a marker for JNK activity. This finding strongly suggests that leptin stimulation broadly activates the JNK signaling pathway in FLSs, resulting in increased JNK expression (**Figure 7A**). Notably, green fluorescence was significantly diminished across all treatment groups (NOB, CB, JNKI). This observation indicates that treatments with NOB, CB, and the JNK inhibitor effectively suppressed JNK expression in FLSs following leptin stimulation.

Compared with the CON control group, high-intensity green fluorescence was observed in the nuclei of FLSs in the LEP group (**Figure 7B**). This indicated that leptin stimulation increased JNK phosphorylation in FLSs, causing a significant nuclear translocation of p-JNK. In contrast, the CB group exhibited a marked decrease in green fluorescence intensity and a significant reduction in p-JNK expression within the FLS nuclei compared to the LEP group. This pattern was mirrored in the JNKI and NOB groups, with no statistically significant differences observed among these three treatment groups. Additionally, the expression of c-Jun, a transcription factor downstream of p-JNK, was assessed.

As shown in **Figure 7C**, c-Jun expression was significantly increased under leptin stimulation in the LEP group. In contrast, both the NOB and the CB groups substantially reversed leptin-induced c-Jun overexpression. The average fluorescence intensity was expressed as the ratio of integrated optical density (IOD) of the target protein to nuclear area (DAPI-stained region), denoted as IOD/Area. Statistical analysis showed that, compared with the control group (CON), the nuclear fluorescence intensity of p-JNK was significantly increased in the leptin (LEP)-treated group (p < 0.01), indicating upregulated JNK phosphorylation and prominent nuclear translocation. After treatment with CB-H or Nobiletin, the nuclear fluorescence intensity of p-JNK was significantly reduced compared to the LEP group (p < 0.01), and this inhibitory effect was not statistically different from that observed in the JNK inhibitor group (SP600125).The degree of inhibition was not significantly different from that observed in the JNKI group. These findings suggest that NOB and CB administration inhibit leptin-induced JNK phosphorylation, p-JNK nuclear translocation, and activation of downstream transcription factors, producing effects similar to those of the JNK inhibitor SP600125.

### 3.8 CB restrains the activation of the JNK/AP-1 pathway in CIA rats

**Figure 8:**
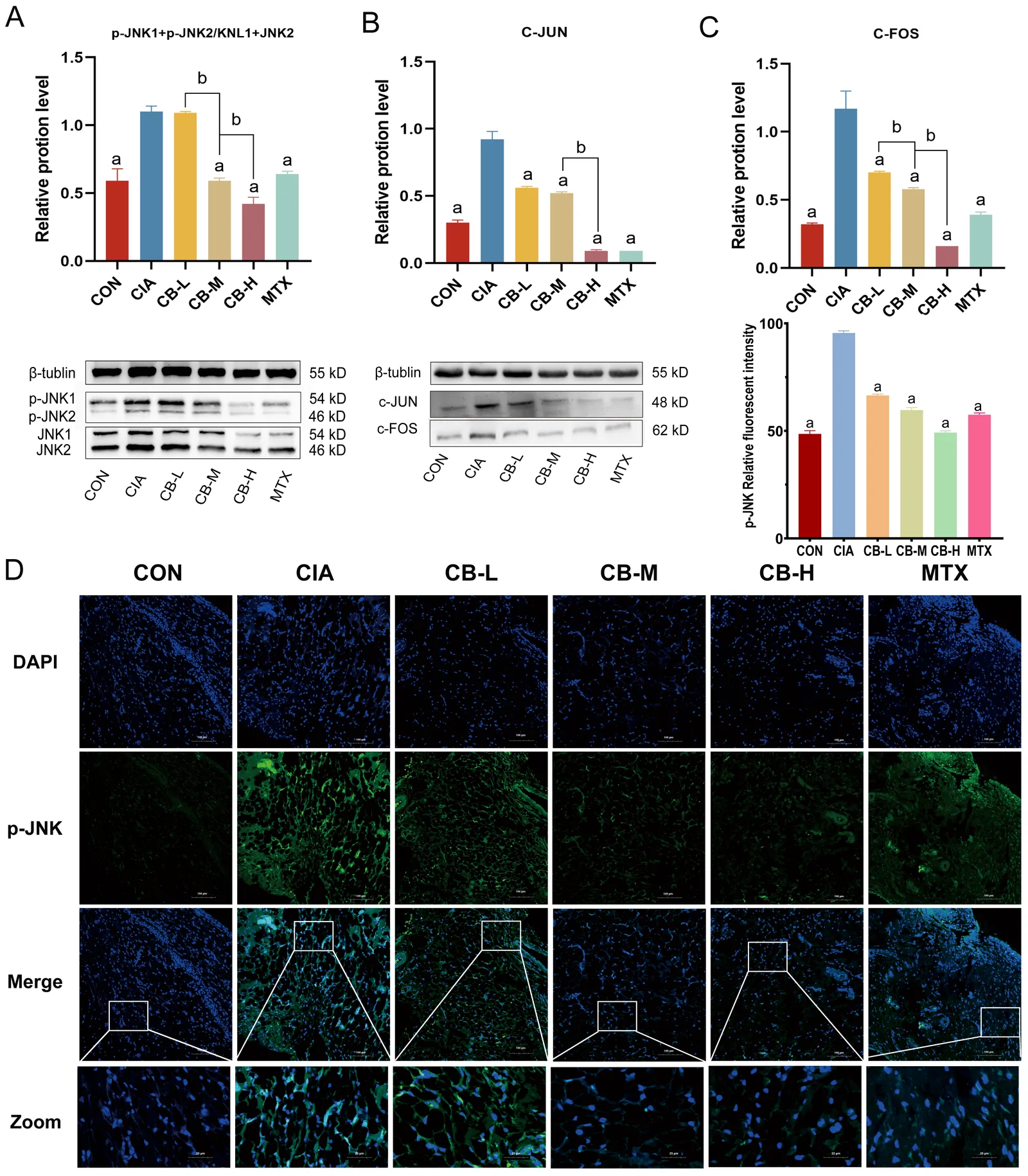
CB inhibits the activation of the JNK pathway in CIA rats (mean ± SD, n = 3). **(A–D)** Expression levels of p-JNK1+JNK2/JNK1+JNK2 **(A)**, c-JUN **(B)**, and c-FOS **(C)** in the ankle joints of CIA rats. **(D)** p-JNK in CIA rat synovium was detected by immunofluorescence in each group. p-JNK (green) and DAPI (blue) (Scale bar = 100 μm; Zoomed scale bar = 25 μm). a: compared with the CIA group, *p* < 0.01; b: compared between two selected groups, *p* < 0.01.

In view of the aforementioned effects of NOB and CB on the JNK/AP-1 pathway in FLS *in vitro*, *in vivo* models were established to investigate the mechanism by which CB modulates synovial inflammation through inhibition of the JNK/AP-1 signaling pathway. WB analysis was performed to assess the effect of CB on the expression of key components of the JNK/AP-1 pathway under different conditions. The ratio of p-JNK1+JNK2/JNK1+JNK2 phosphorylation and the expression levels of c-JUN and c-FOS were significantly higher in the ankle joints of CIA rats compared to the CON group (*p* < 0.01, **Figures 8A–C**). Phosphorylation levels of p-JNK1+JNK2/JNK1+JNK2 and the expression of c-JUN and c-FOS in the rats’ ankle joints were significantly reduced in a dose-dependent manner following treatment with CB and MTX (*p* < 0.01).

Further, similar results were obtained by observing the activation of the JNK/AP-1 pathway in the synovial membranes of CIA rats (**Figure 8D**). p-JNK was highly expressed in the synovial tissues of rats in the CIA group. Similarly, p-JNK was highly expressed in both the nuclei and cytoplasm of FLS cells. Compared with the CON group, the CIA group exhibited high phosphorylation of JNK and enhanced p-JNK nuclear translocation in the synovium of CIA rats. As expected, the expression of p-JNK in rat synovium decreased in a dose-dependent manner with increasing concentrations of CB. P-JNK expression was partially inhibited in the MTX group but the expression level of p-JNK was higher in the MTX group than in the CB-H group. These results suggest that the JNK signaling pathway is abnormally activated in RA and that CB can inhibit its expression.

## 4 Discussion

RA is a systemic autoimmune disease characterized by persistent synovial inflammation, cartilage destruction, and bone erosion (1, 28). Although current therapies have substantially improved disease control, some patients still show inadequate or partial treatment responses, reflecting the clinical heterogeneity and mechanistic complexity of RA (29–32). Traditional Chinese medicines contain multiple bioactive constituents that may act on interconnected targets and signaling pathways, providing a potential complementary strategy for RA management (33, 34). In the present study, CB significantly reduced paw swelling and arthritis scores and ameliorated synovial inflammation, cartilage damage, and bone destruction in CIA rats. These findings support the anti-arthritic effects of CB in this experimental model.

CB is a classic herbal pair widely used in traditional Chinese medicine for disorders associated with “internal dampness.” Chenpi, derived from dried citrus peel, has been reported to exert anti-inflammatory, lipid-lowering, and antioxidant effects (15). Several Chenpi-derived constituents have also shown beneficial effects in experimental RA (35, 36). In particular, nobiletin has been reported to reduce angiogenesis and inflammation in the synovial tissues of CIA rats by inhibiting the p38/NF-κB signaling pathway (37). Banxia, the dried tuber of *Pinellia ternata* (Thunb.), is traditionally used in Chinese medicine to dispel dampness and eliminate phlegm (38, 39). Pharmacological studies have shown that Banxia possesses a range of effects, including antitussive, expectorant, antiemetic, antitumor, and anti-inflammatory properties (40–42). Some studies have reported that Banxia’s total alkaloids exhibit significant anti-inflammatory effects, influencing the release and inhibition of inflammatory factors (43–45). Additionally, Banxia affects the expression of inflammation-related pathways, including the TNF signaling pathway, PI3K/Akt signaling pathway, and Rat sarcoma virus (Ras) signaling pathway (46, 47). In the present study, CIA rats were used as models to investigate the mechanism of action of CB in treating RA. Our results confirmed the therapeutic effects of CB on RA in CIA rats, consistent with previous studies. Specifically, CB treatment significantly reduced paw swelling, arthritis score, while also delaying disease progression in the CIA rats. Histopathological analysis revealed that CB treatment reduced pathological scores and improved joint pathology in CIA rats.

The pathophysiological mechanisms of RA remain unclear (48, 49). Recently, numerous studies have explored the underlying mechanisms of TCM in treating diseases through network pharmacology combined with molecular docking (33, 42, 50, 51). In this study, network pharmacology analysis, molecular docking, and HPLC were used to investigate the underlying mechanism of action of CB. A total of 17 active compounds and 134 potential CB targets were identified. Topological analysis of the PPI network revealed 9 hub genes. GO enrichment analysis indicated that CB exerts its anti-RA effects by modulating synoviocyte proliferation, growth, and apoptosis. Notably, CB targets were found to be closely correlated with genes regulating lipid metabolism and nutrient levels. Furthermore, KEGG pathway enrichment analysis identified 20 enriched pathways, among which the JNK signaling pathway—implicated in lipid metabolism regulation—was significantly enriched. Molecular docking and molecular dynamics (MD) simulations revealed that the primary active constituents of the CB herb pair—nobiletin (derived from Chenpi) and β-sitosterol (derived from Banxia)—both exhibit robust binding affinities toward core targets within the JNK signaling pathway. Consequently, the CB herb pair likely alleviates rheumatoid arthritis (RA) by modulating the JNK pathway and suppressing its downstream inflammatory responses. Although our computational predictions indicated that both components possess high binding energies with key JNK pathway proteins, our preliminary screening of the five primary bioavailable constituents in serum demonstrated that the semi-quantitative peak area response of nobiletin was markedly higher than that of β-sitosterol, suggesting a higher systemic exposure of nobiletin *in vivo*. Furthermore, nobiletin is a characteristic polymethoxyflavone that serves as a distinctive chemical marker for Chenpi, whereas β-sitosterol is a ubiquitous compound found across numerous botanicals rather than being specific to Pinellia ternata (Banxia). Therefore, this study prioritized the further validation of the interaction between nobiletin and JNK. We hypothesize that β-sitosterol may act on upstream regulators or downstream effectors of the JNK pathway, thereby exerting a ‘multi-component, common-target’ synergistic amplification effect with nobiletin—a mechanism we intend to investigate in future studies.

**Figure 9:**
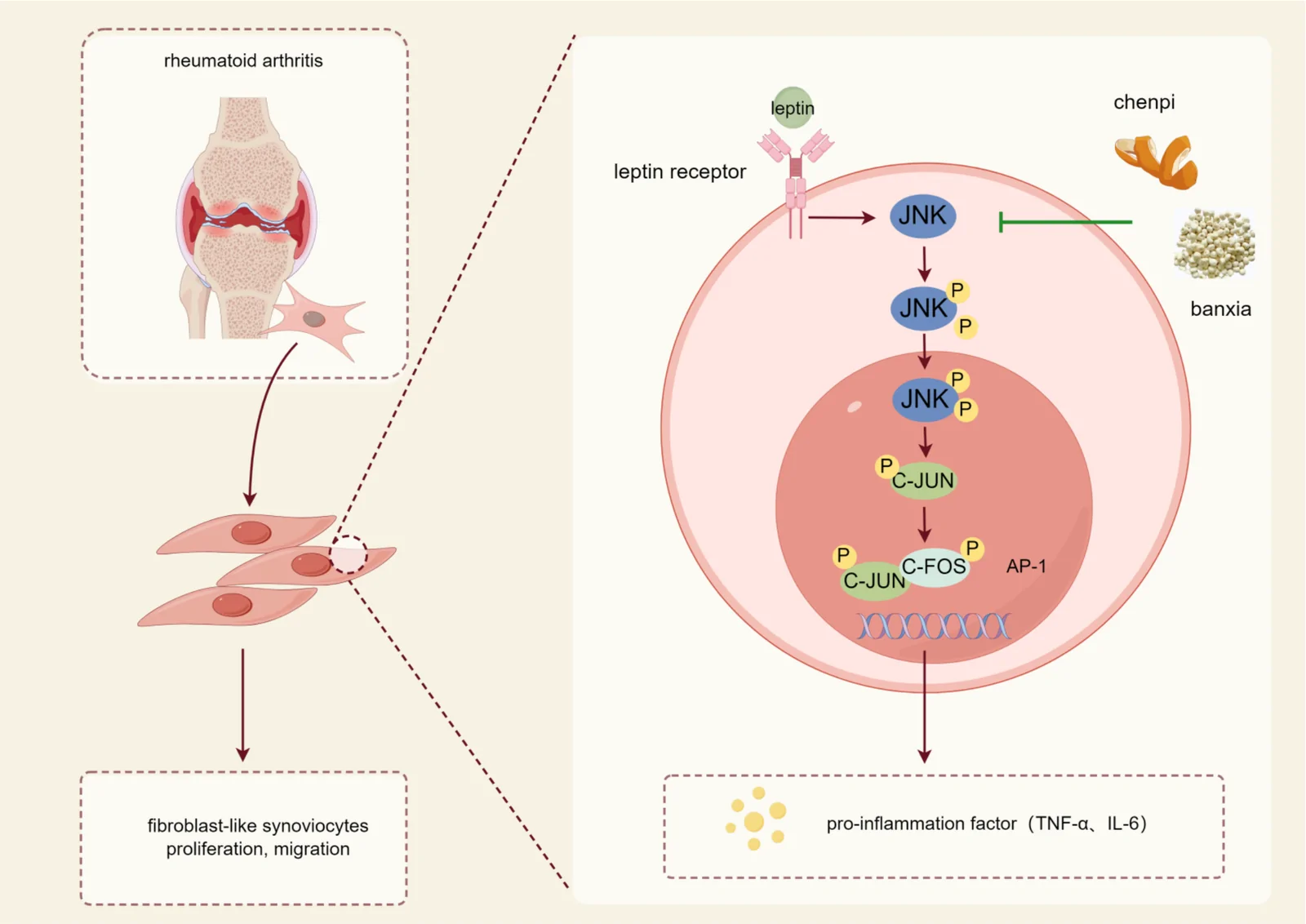
A summary of the mechanisms underlying the effects of CB in RA.

FLS play key roles in the regulation of the proliferation and inflammation of RA synovial tissue (52), owing to their processes, such as the proliferation, migration, adhesion, invasion, and secretory functions. Research has demonstrated that FLSs may participate in the leptin-mediated RA pathophysiology (53). Furthermore, leptin exacerbates the occurrence of oxidative stress (OS) in RA-FLS, intensifying inflammatory responses, cell migration, and angiogenesis in Human Umbilical Vein Endothelial Cells (HUVECs) (54). Therefore, alleviating the leptin-mediated functional abnormalities in RA-FLSs may be an attractive strategy to control RA synovial inflammation and growth, thereby preventing bone and cartilage erosion, and improving RA outcomes. Various signaling pathways regulate RA-FLSs, including the JNK/AP-1 signaling pathway, which is a well-established mechanism in RA pathogenesis (55). The JNK/AP-1 signaling pathway, which involves the transcription factors c-Jun and c-Fos, plays a crucial role in various inflammatory diseases. AP-1, a key transcription factor composed of c-Fos and c-Jun, regulates the expression of inflammation-related genes. The proposed mechanism by which CB alleviates synovial inflammation through modulation of the leptin-mediated JNK/AP-1 signaling pathway is summarized in **Figure 9**. This pathway involves the phosphorylation of JNK, c-Jun, and c-Fos, leading to the formation of c-Jun/c-Fos heterodimers, which directly promote the transcription of inflammation factors. Inhibition of the JNK/AP-1 pathway has been shown to suppress inflammation, highlighting its role as an upstream regulator of inflammatory responses. For instance, JNK act downstream of pro-inflammatory cytokines such as leptin and TNF-α. Activation of JNK by upstream kinases and phosphatases contributes to the synovial cell inflammation (56, 57). Addressing the over-activation of the JNK signaling pathway in RA may be crucial for mitigating RA-FLSs pathological responses. However, single-target JNK inhibitors have been associated with risks, including increased cardiovascular risk and hepatotoxicity (58, 59). Therefore, agents that inhibit the JNK signaling while minimizing the above risks is essential.

Evidence has shown that FLS participates in the pathogenesis of RA (60, 61). FLS in the synovial fluid of the joints become excessively migratory, invasive, and proliferative (62), producing pro-inflammatory cytokines that mediate both the inflammatory response and joint destruction (63, 64). These cells regulate arthritis inflammation by producing diverse cytokines (e.g., IL-6 and TNF-α), pro-angiogenic factors (e.g., vascular endothelial growth factor), and chemokines (e.g., CXC-chemokine ligand 10) (65). Among these, IL-6 and TNF-α are crucial regulators of inflammation and tissue injury in RA joints (66–68). IL-6 promotes inflammatory joint destruction in RA (69), and Lee and Yoo (70) confirmed that TNF-α signaling persists in RA-FLSs, leading to an uncontrolled synovial inflammatory response. TNF-α stimulation, in turn, sustains synovial inflammation in RA (71). Therefore, IL-6 and TNF-α in rat serum, synovium, and FLS are potential biomarkers of inflammatory activation in RA. In the present study, we found that IL-6 and TNF-α were highly expressed in the serum and synovium of CIA rats. However, CB treatment dose-dependently reduced their expression levels. A similar trend was observed in leptin-stimulated FLSs. In these cells, IL-6 and TNF-α expression was significantly elevated in the LEP group. Yet, both CB-containing serum and nobiletin effectively inhibited the expression of IL-6 and TNF-α, with CB-containing serum showing a dose-dependent effect. These results suggest that CB and nobiletin can exert anti-RA effects by inhibiting the release of multiple inflammatory factors in both CIA rats and leptin-stimulated FLSs. Furthermore, CB appears to downregulate a broad spectrum of inflammatory cytokines, offering potential therapeutic advantages over current single-target biologics.

Evidence from numerous studies has demonstrated that the biological properties of FLSs are altered by inflammatory factors in RA patients with synovitis (61, 72). This alteration leads FLSs to exhibit multiple tumor-like behaviors, including hyperproliferation, invasion, and increased migration (73). Therefore, inhibiting the proliferation and migration of RA-FLSs may be an attractive strategy for improving RA. In this study, we investigated the effects of CB on FLSs proliferation and migration using a leptin-stimulated FLS model. The results showed that leptin, at a concentration of 100 ng/mL, significantly enhanced the proliferation and migration of FLSs. In contrast, both CB-containing serum and 25 uM nobiletin, at medium and high doses, significantly reduced leptin-induced FLSs proliferation and migration.

JNK belongs to the MAPK family (74). The JNK/AP-1 pathway is primarily activated in response to oxidative stress. The JNK signaling pathway plays key roles in development, apoptosis, cell growth, inflammation, and immune responses (75). Previous studies have detected increased JNK phosphorylation in joint extracts from CIA mice (38, 76, 77), and JNK activation has also been observed in rats with adjuvant-induced arthritis (AIA) (78). The JNK signaling pathway can be activated by various upstream effectors, and phosphorylated JNK regulates several cellular processes, including cell differentiation, proliferation, and survival. These processes play distinct roles in different types of diseases (75). Kim et al. found that the JNK-specific inhibitor SP600125 significantly reduced foot swelling, joint damage, and the levels of inflammatory factors in AIA rats (79). Collectively, these studies demonstrate that the JNK/AP-1 pathway is activated in RA, and inhibition of JNK/AP-1 activation improves RA symptoms by alleviating synovial inflammation (39, 80, 81), consistent with the findings of the present study. CB inhibited the activation of the JNK/AP-1 pathway in CIA rats by reducing JNK phosphorylation and decreasing the expression of c-Jun and c-Fos. Thus, CB improved RA by inhibiting aberrant leptin-mediated activation of the JNK/AP-1 signaling pathway.

However, several limitations of this study should be acknowledged. As a preliminary exploration focused on the CB herb pair, this study only partially elucidates the anti-RA efficacy and underlying molecular mechanism of a single monomer, nobiletin. Traditional Chinese medicine typically exerts therapeutic effects through complex, multi-component, and multi-target cooperative networks. Consequently, the independent therapeutic contributions of other primary constituents—particularly the active alkaloids from Pinellia ternata (Banxia) and remaining flavonoids from Citrus reticulata (Chenpi)—as well as their potential “multi-component, common-target” synergistic amplification effects with nobiletin, remain to be fully characterized. Furthermore, these findings are restricted to animal and *in vitro* models, lacking clinical validation and long-term safety profiles in RA patients. Future research will progressively investigate the molecular synergy among these co-existing components to fully decode the complex mechanism of this herb pair in clinical translation.

## 5 Conclusions

In summary, this study demonstrated the therapeutic potential of CB in RA. CB significantly alleviated RA symptoms in CIA rats, reducing histopathological scores and the levels of IL-6 and TNF-α in both serum and synovium. Using network pharmacology, this study constructed an interaction network between CB and RA, identifying multiple active components such as nobiletin that might underlie CB’s therapeutic effects. HPLC analysis quantified nobiletin as the most abundant active component of CB in the serum. Molecular docking and MD analysis suggested that the JNK signaling pathway could be involved in its action. *In vitro* experiments further confirmed that nobiletin inhibited the inflammation, proliferation, and migration of FLS through the leptin - mediated JNK/AP-1 signaling pathway. Finally, CB-containing serum significantly downregulated the expression of the JNK/AP-1 signaling pathway in CIA rat models, thereby achieving its therapeutic effects. Overall, these findings establish a scientific basis for the potential use of CB in preventing and treating RA, suggesting that CB could be a promising therapeutic option for RA.

## Data Availability

The original contributions presented in the study are included in the article/**Supplementary Material**. Further inquiries can be directed to the corresponding authors.

## Supplementary material

Supplementary Figure 1 CCK-8 assay for screening the optimal concentration of the JNK inhibitor (JNKI).

Supplementary Figure 2 CCK-8 assay for screening the optimal concentration of nobiletin (NOB).

Supplementary Figure 3 CCK-8 assay for screening the optimal concentration of leptin.

Supplementary Figure 4 CCK-8 assay for screening the optimal concentration of CB-containing serum.
