# Supplementary material for: Combination of Chenpi and Banxia alleviates synovial inflammation in a rat model of rheumatoid arthritis by targeting the JNK/AP-1 signaling pathway
Source: Front Immunol. 2026 Sep 11;17:1889598. doi: 10.3389/fimmu.2026.1889598 (PMC13612294; doi:10.3389/fimmu.2026.1889598)
Supplement: Supplementary file 1 [file Table1.docx]

Figure 6J：

β-tublin

p-JNK1 p-JNK2

JNK1JNK2：

Figure6K：

β-tublin：

c-Jun：

c-FOS：
